# The Circumferential Resection Margin Is a Prognostic Predictor in Colon Cancer

**DOI:** 10.3389/fonc.2020.00927

**Published:** 2020-06-26

**Authors:** Xin-Yi Tang, Meng-Xi Huang, Si-Qi Han, Yue Chang, Zhi-Ping Li, Xiao-Ming Kao, Yan-Yan Chen, Chao Liu, Ya-Di Huang, Yi-Tian Chen, Zeng-Jie Lei, Xiao-Yuan Chu

**Affiliations:** ^1^Department of Medical Oncology, School of Medicine, Jingling Hospital, Nanjing University, Nanjing, China; ^2^School of Medicine, Nanjing University, Nanjing, China; ^3^Department of Medical Oncology, Jinling Hospital, Nanjing Clinical School of Southern Medical University, Nanjing, China; ^4^Department of General Surgery, Jinling Hospital, Medical School of Nanjing University, Nanjing, China; ^5^Department of Medical Oncology, Jinling Hospital, Nanjing Clinical School of Nanjing Medical University, Nanjing, China

**Keywords:** circumferential resection margin, cutoff, colon cancer, prognostic predictor, SEER database

## Abstract

**Objective:** This study aimed to investigate the potential value of circumferential resection margin (CRM) in colon cancer prognostics.

**Summary Background Data:** CRM has been extensively studied as an important prognostic factor in rectal and esophageal cancer, but not in colon cancer.

**Methods:** Data from 6,681 CRM-positive patients and 25,908 CRM-negative patients diagnosed with colon cancer in 2010–2015 were obtained from the Surveillance, Epidemiology, and End Results database. Statistical analysis methods utilized included the chi-square test, Kaplan-Meier estimates, Cox proportional, and X-tile software analyses.

**Results:** After propensity score matching, CRM positivity was found to be negatively related with survival *(P* < 0.001). X-tile software identified 0 and 30 mm as optimal cutoff values (*P* < 0.001) for prognosis, which was applicable only in stage II–IV patients. A 20 and 33% risk decrease were observed in patients with CRM between 0 and 30 mm [95% confidence interval (CI) = 0.76–0.84], and larger than 30 mm (95% CI = 0.62–0.71), respectively. Chemotherapy strongly benefited prognosis with a hazard ratio of 0.36 (95% CI = 0.34–0.38) for overall survival (OS). Patients with a CRM value of 0–30 mm seemed to benefit most from chemotherapy compared with other groups. CRM and number of regional lymph nodes are independent risk factors, and the latter is a good substitute for CRM in AJCC stage I patients.

**Conclusion:** CRM positivity is a strong unfavorable survival indicator for colon cancer patients. A better outcome is expected with CRM values larger than 30 mm. This cutoff value only applied to stage II–IV patients. For stage I patients, number of regional lymph nodes is a good substitute to predict survival. Chemotherapy was another favorable prognostic factor, especially for patients with a CRM value between 0 and 30 mm.

## Introduction

Colorectal cancer is the third most prevalent carcinoma in both males and females in the United States, and is mainly treated by surgery, chemotherapy, as well as radiation (1). As the main treatment for colorectal cancer, surgery is expected to minimize local recurrence and prolong disease-free survival. For optimum results, it is critical to ensure no tumor invasion at the edge of the specimen. The circumferential resection margin (CRM), is a term used to describe the relationship between the resection margin and the tumor. According to the 8th Edition of the American Joint Committee on Cancer (AJCC) Cancer Staging Manual, the CRM refers to the distance in millimeters between the deepest point of tumor invasion in the primary cancer and the margin of resection in the retroperitoneum or mesentery (2). Pathologically, CRM involvement (also called CRM positivity) should be defined as presence of remnant tumor cells after resection (3).

As early as 1986, CRM involvement was suggested to be a prognostic factor for recurrence in rectal cancer (4), which was later confirmed by additional studies (5). In 1993, the concept of CRM was introduced to the esophageal adenocarcinoma field (6), and was later verified to hold some prognostic value (7, 8). Since then, CRM positivity continued to be studied, with different opinions arising about its prognostic significance, varying mainly due to the application of modern multidisciplinary management (9) and neoadjuvant therapy (10, 11). Additionally, incremental benefits in survival and recurrence rates with increasing CRM values have been found in esophageal cancer (12).

For colon cancer, the significance of CRM remains to be investigated. The 2015 study by Amri et al., which included data from 984 patients (52 of them were CRM positive), was the first to confirm the prognostic significance of CRM involvement in colon cancer, and showed that CRM positivity is a stage-independent outcome predictor linked with higher rates of recurrence and worse survival (13). However, this study failed to reach statistical significance for some of the outcomes because of a small sample size.

In this retrospective study, we investigated the association of CRM positivity with prognosis in colon cancer in a large sample size. We also explored whether there was a significant difference in survival among subgroups of CRM-negative patients, or whether these patients could be divided into subgroups according to prognosis. In addition, we investigated optimal CRM value cutoffs for different AJCC stages separately, since there is a strong influence of AJCC stage on outcomes, to verify its application in colon cancer at different stages. Lastly, we hoped to identify risk factors associated with colon cancer survival and whether the effects of other strong predicators would be influenced by CRM values.

## Materials and Methods

### Data Source and Inclusion Criteria

The Surveillance, Epidemiology, and End Results (SEER) database is an authoritative source for cancer statistics in the United States, where it collects information about cancer patients in 18 registries and covers about 30% of the total U.S. population (14). Data from patients diagnosed with colon cancer and who underwent surgery between 2010 and 2015 were obtained from the database. Supplementary Figure 1 depicts the data selection process, which resulted in the inclusion of 6,681 CRM-positive patients and 25,908 CRM-negative patients for analysis.

### Statistical Analysis

All analyses were performed using the R statistical software (version 3.5.0) by Bell Laboratories in New Jersey, United States. Demographic and clinical features were analyzed with a chi-square (χ^2^) test. A two-sided *P* < 0.05 was considered statistically significant. In order to adjust for differences in baseline characteristics and to minimize bias, propensity score matching with a ratio of 1:1 was performed with the “MatchIt” R package. Survival curves were generated using Kaplan-Meier estimates. The X-tile program was used to generate the optimal CRM cutoff points with minimum *P* values from chi-square tests (15). Both univariate and multivariate Cox proportional hazard models were used to identify variables associated with survival, and the results are presented as hazard ratios (HRs) and 95% confidence intervals (95% CIs). The endpoints for this study were overall survival (OS) and cancer-specific survival (CSS). Patients who were alive at the time of the last follow-up were censored.

## Results

### CRM Positivity Is an Unfavorable Factor for Survival

**Figure 1:**
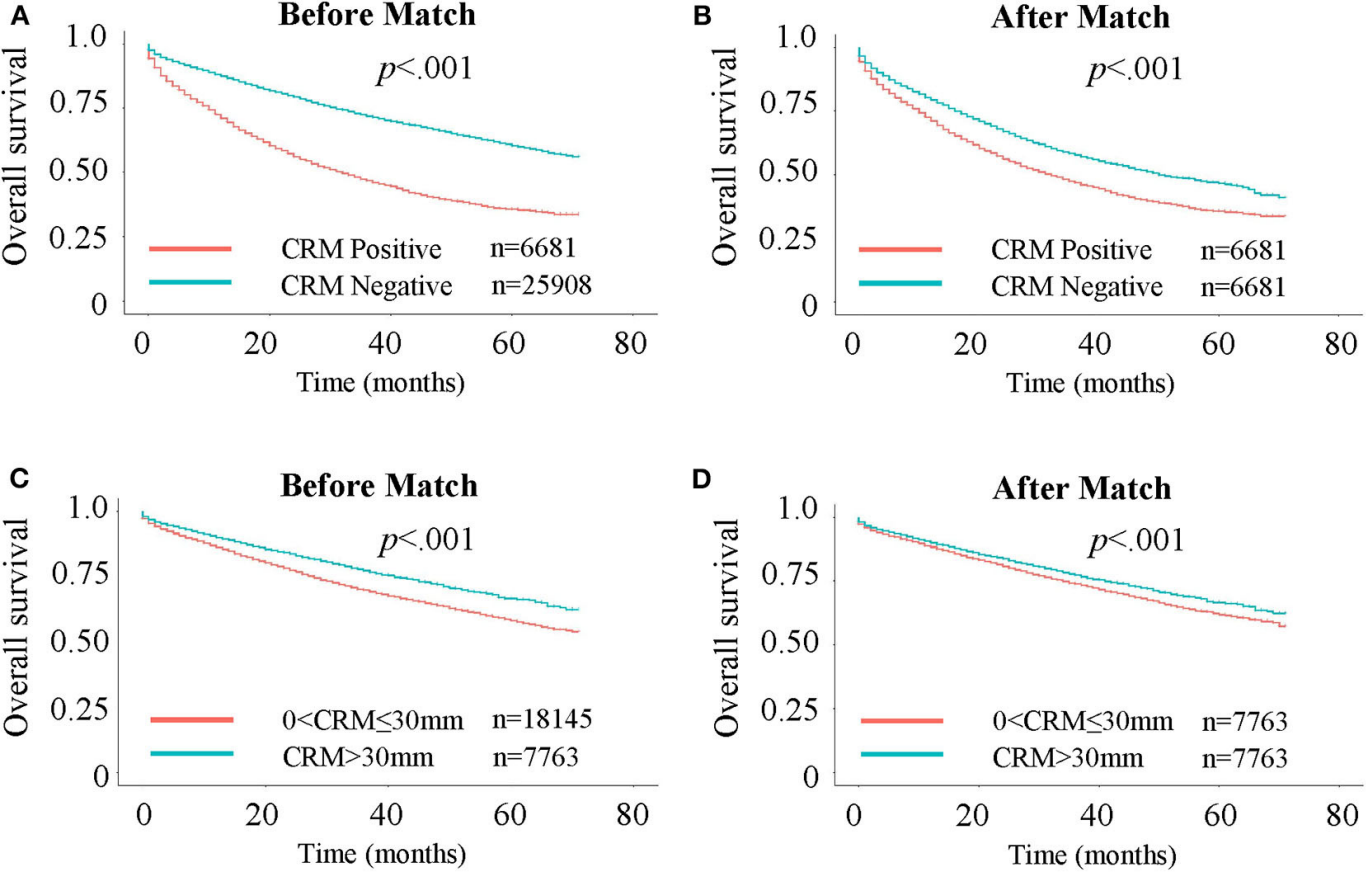
Kaplan-Meier curves for Overall Survival (OS). **(A)** OS in CRM-positive/negative patients. **(B)** OS in CRM-positive/negative patients after 1:1 propensity score matching. **(C)** OS in patients with CRM values of 0–30 and >30 mm. **(D)** OS in patients with CRM values of 0–30 and >30 mm after 1:1 propensity score matching.

A total of 32,589 eligible patients were included in this study, with 6,681 (20.5%) patients being CRM-positive and 25,908 (79.5%) patients being CRM-negative. The demographic and clinical features of the two cohorts are shown in Table 1. Significant differences were observed between these two groups. CRM status was shown to be a significant unfavorable predictor for both OS (Figure 1A, *P* < 0.001) and CSS (Supplementary Figure 2A, *P* < 0.001), as assessed by a Kaplan-Meier analysis.

**Table 1:** Baseline characteristics for CRM-positive and CRM-negative patients prior to and after propensity score matching.

**Variables**	**Before matching**			**After matching**		

	**CRM positive (*****n*** **=** **6,681)**	**CRM negative (*****n*** **=** **25,908)**	***p***	**CRM positive (*****n*** **=** **6,681)**	**CRM negative (*****n*** **=** **6,681)**	***p***
	**n (%)**	**n (%)**		**n (%)**	**n (%)**	
Age			<0.001			0.647
<65	2,709	9,361		2,709	2,682	
	(40.55)	(36.13)		(40.55)	(40.14)	
≥65	3,972	16,547		3,972	3,999	
	(59.45)	(63.87)		(59.45)	(59.86)	
Sex			0.288			0.177
Male	3,261	12,837		3,261	3,340	
	(48.81)	(48.55)		(48.81)	(49.99)	
FeMale	3,420	13,071		3,420	3,341	
	(51.19)	(50.45)		(51.19)	(50.01)	
Race			0.019			0.084
White	5,405	20,818		5,405	5,304	
	(80.9)	(80.35)		(80.9)	(79.39)	
Black	753	2,794		753	802 (12)	
	(11.27)	(10.78)		(11.27)		
Other (American Indian/	523	2,296		523	575	
AK Native, Asian/	(7.83)	(8.86)		(7.83)	(8.61)	
Pacific Islander)						
Year of diagnosis			<0.001			<0.001
2010	1,201	3,288		1,201	898	
	(17.98)	(12.69)		(17.98)	(13.44)	
2011	1,159	4,028		1,159	1,039	
	(17.35)	(15.55)		(17.35)	(15.55)	
2012	1,149	4,217		1,149	1,106	
	(17.2)	(16.28)		(17.2)	(16.55)	
2013		4,383		1,030	1,094
	(15.42)	(16.92)		(15.42)	(16.37)	
2014	1,151	4,867		1,151	1,251	
	(17.23)	(18.79)		(17.23)	(18.72)	
2015	991	5,125		991	1,293	
	(14.83)	(19.78)		(14.83)	(19.35)	
AJCC^a^			<0.001			0.980
I	484	5,425		484	488	
	(7.24)	(20.94)		(7.24)	(7.3)	
II	1,719	9,069		1,719	1,720	
	(25.73)	(35)		(25.73)	(25.74)	
III	2,431	8,453		2,431	2,409	
	(36.39)	(32.63)		(36.39)	(36.06)	
IV	2,047	2,961		2,047	2,064	
	(30.64)	(11.43)		(30.64)	(30.89)	
T^a^			<0.001			<0.001
T1	303	2,444		303	303	
	(4.54)	(9.43)		(4.54)	(4.54)	
T2	293	4,132		293	520
	(4.39)	(15.95)		(4.39)	(7.78)
T3	2,704	15,643		2,704	4,336	
	(40.47)	(60.38)		(40.47)	(64.9)	
T4	3,381	3,689	3,381	1,522	
	(50.61)	(14.24)		(50.61)	(22.78)	
N^a^			<0.001			<0.001
N0	2,427	15,000		2,427	2,453
	(36.33)	(57.9)		(36.33)	(36.72)	
N1	1,909	6,899		1,909	2,085	
	(28.57)	(26.63)		(28.57)	(31.21)	
N2	2,345	4,009		2,345	2,143	
	(35.1)	(15.47)		(35.1)	(32.08)	
M^a^			<0.001			0.764
M0	4,634	22,947		4,634	4,617	
	(69.36)	(88.57)		(69.36)	(69.11)	
M1	2,047	2,961		2,047	2,064	
	(30.64)	(11.43)		(30.64)	(30.89)	
Site^b^			<0.001			0.114
Right colon	4,050	16,733		4,050	4,140	
	(60.62)	(64.59)		(60.62)	(61.97)	
Left colon	2,631	9,175		2,631	2,541
	(39.38)	(35.41)		(39.38)	(38.03)	
Histology^c^			<0.001			0.213
Adenocarcinoma	5,723	20,998		5,723	5,671	
	(85.66)	(81.05)		(85.66)	(84.88)	
Non-adenocarcinoma	958	4,910		958	1,010	
	(14.34)	(18.95)		(14.34)	(15.12)	
Surgery			0.888			0.126
Partial colectomy	2,513	9,792		2,513	2,607	
	(37.61)	(37.8)		(37.61)	(39.02)	
Subtotal/ Hemicolectomy	4,013	15,551		4,013	3,946	
	(60.07)	(60.02)		(60.07)	(59.06)	
Total colectomy	135	486		135	116	
	(2.02)	(1.88)		(2.02)	(1.74)	
Total proctocolectomy	20	79		20	12
	(0.3)	(0.3)		(0.3)	(0.18)
Radiation			<0.001			<0.001
No/unknown	6,410	25,606		6,410	6,561	
	(95.94)	(98.83)		(95.94)	(98.2)	
Yes	271	302		271	120	
	(4.06)	(1.17)		(4.06)	(1.8)	
Chemotherapy			<0.001			0.150
No/unknown	3,650	17,261		3,650	3,566	
	(54.63)	(66.62)		(54.63)	(53.38)	
Yes	3,031	8,647		3,031	3,115	
	(45.37)	(33.38)		(45.37)	(46.62)	
Regional LN examined			<0.001			0.376
0	68	92		68	72
	(1.02)	(0.36)		(1.02)	(1.08)	
LN <12	1,170	2,507		1,170	1,100	
	(17.51)	(9.68)		(17.51)	(16.46)	
12 ≥ LN <24	3,995	16,187		3,995	4,074	
	(59.8)	(62.48)		(59.8)	(60.98)	
LN ≥ 24	1,448	7,122		1,448	1,435	
	(21.67)	(27.49)		(21.67)	(21.48)	
Regional LN positive			<0.001			0.009
No/unknown	2,510	15,414		2,510	2,535	
	(37.57)	(59.5)		(37.57)	(37.94)	
LN <6	2,391	7,988		2,391	2,471	
	(35.79)	(30.83)		(35.79)	(36.99)	
6 ≥ LN <12	1,040	1,799		1,040	1,052	
	(15.57)	(6.94)		(15.57)	(15.75)	
LN ≥ 12	672	615		672	551	
	(10.06)	(2.37)		(10.06)	(8.25)	
No LN Examined	68 (1.02)	92 (0.36)		68 (1.02)	72 (1.08)	

a *American Joint Committee on Cancer (16)*.

b *Right colon included cecum, ascending colon, hepatic flexure, and transverse colon, while left colon included splenic flexure, descending colon, and sigmoid colon*.

c *Adenocarcinoma included International Classification of Diseases for Oncology = 8,140, 8,201, 8,213, 8,260, 8,480, 8,490, 8,510*.

To further assess the impact of CRM status on survival, a bipartite propensity score matching analysis was performed with stratification for age, AJCC stages (16), administration of chemotherapy, number of regional lymph nodes examined and positive regional lymph nodes. After matching, significant differences were only observed for year of diagnosis, T and N stages, administration of radiation, and number of positive regional lymph nodes, thus minimizing the possible bias from different baseline characteristics when analyzing the effect of CRM on survival (Table 1 after matching). After propensity score matching, CRM-negative patients still had more favorable outcomes in both OS (Figure 1B, *P* < 0.001) and CSS (Supplementary Figure 2B, *P* < 0.001).

### Optimal CRM Value Cutoffs

**Figure 2:**
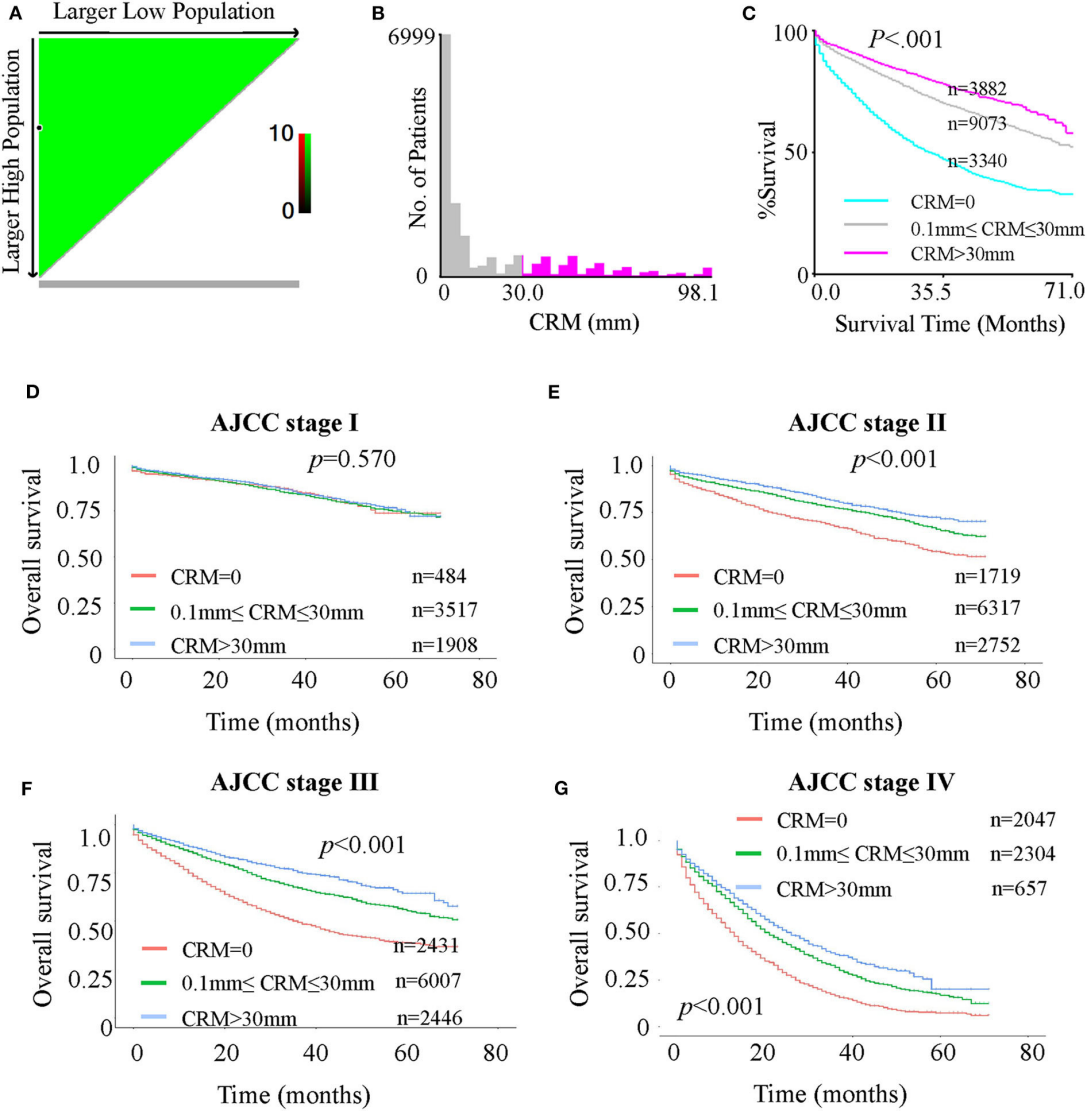
(A-C): X-tile analysis of survival data for optimal CRM cutoff values from records of all patients. **(A)** Continuous direct association (colored in green) between increasing CRM values and improved survival, identifying 0 and 30 mm as the optimal cutoff values using the minimum *p*-value method. **(B)** Distribution of CRM values among patients. **(C)** The minimal *P*-value determined by the optimal cutoff values of 0 and 30 mm (*P* < 0.0001). (D-G): Kaplan-Meier curves for OS in patients with different AJCC stages stratified by CRM records. **(D)** OS in stage I patients with CRM values of 0, 0–30, and >30 mm. **(E)** OS in stage II patients with CRM values 0 mm, 0–30 and > 30 mm. **(F)** OS in stage III patients with CRM values 0, 0–30, and >30 mm. **(G)** OS in stage IV patients with CRM values 0, 0–30, and > 30 mm.

In addition to confirming that CRM positivity is a strong unfavorable prognostic indicator, we aimed to explore the value of further dividing CRM-negative patients into different subgroups. Analyses using the X-tile program identified 0 and 30 mm as the optimal cutoff values (Figure 2). In this study, we divided the cohort with two cut-points, producing high, medium, and low subsets, and patient data from the SEER registry were equally divided into training and validation sets in a randomized way. In the training set, the X-tile program firstly identified possible optimal cutoffs (0 and 30 mm) using the minimum *P*-value method. Figure 2A shows a continuous direct association between increasing CRM value and improved survival since the whole plot is in green, and the black circle indicates the possible optimal cutoffs with minimal *P*-value (0 and 30 mm). Figure 2B shows the distribution of CRM value among patients. Figure 2C is a Kaplan-Meier plot for the three subgroups divided by the two cutoffs. Then, in the validation set, X-tile program tested the significance of *P*-value of such cutoffs and got the result of *P* < 0.0001. In short, the optimal cutoff value highlighted by the black circle in Figure 2A is shown on a histogram of the entire cohort (Figure 2B) as well as a Kaplan-Meier plot (Figure 2C). The figures show patients were divided at the optimal cutoff values of 0 and 30 mm (*P* < 0.0001).

Considering the possible deviation in optimal cutoffs of CRM values due to tumor progression, verification of the optimal cutoff values from all patients to separate AJCC stages is necessary. Kaplan-Meier analyses showed that for both OS (Figures 2 DEFG) and CSS (Supplementary Figure 3), the survival of AJCC stage I patients was not significantly different among the three subgroups (CRM value = 0 mm, 0 mm < CRM value ≤ 30 mm and CRM value > 30 mm). However, a significant difference was observed for stage II–IV patients. The plots suggested that the optimal value cutoffs derived from all patients may not apply to stage I patients. To further investigate this problem, an X-tile analysis for the four separate stages was done (Supplementary Figure 4). A cutoff value with a statistically significant difference among subgroups was not found for stage I patients (Supplementary Figure 4A), while the optimal cutoff values of 0 and 30 mm were found for stage II patients (Supplementary Figure 4B), 0 and 28 mm for stage III patients (Supplementary Figure 4C), and 0 and 33 mm for stage IV patients (Supplementary Figure 4D). In general, 0 and 30 mm were the best cutoff values for stage II–IV patients, but not for stage I patients.

### HRs of Subgroups Divided by CRM Cutoff Values

To assess the exact risk ratio differences for survival between the CRM subgroups, both univariate and multivariate Cox proportional hazard analyses were performed to identify risk factors associated with colon cancer prognosis. For OS (Supplementary Table 1), a 20% and 33% decrease in risk were shown in patients with CRM values between 0 and 30 mm (95% CI = 0.76–0.84) and patients with CRM values larger than 30 mm (95% CI = 0.62–0.71), respectively. Having received radiation therapy was not independently associated with survival, while chemotherapy was demonstrated to strongly benefit prognosis (HR = 0.36, 95% CI = 0.34–0.38). Similar results were achieved for CSS analyses (Supplementary Table 2).

### Patients With a CRM Value Between 0 and 30 mm Benefit Most From Chemotherapy

**Figure 3:**
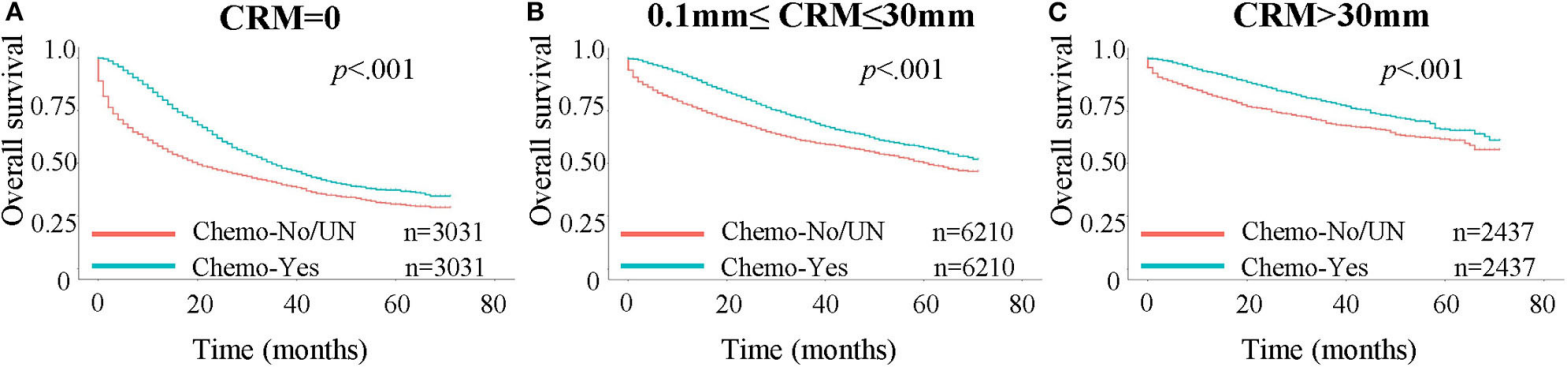
Kaplan-Meier curves for OS in patients stratified by chemotherapy status. A 1:1 propensity score matching was performed, with stratification for age, AJCC stages, number of regional lymph nodes examined and positive regional lymph nodes. Chemo: chemotherapy; UN: unknown. **(A)** OS in patients with CRM values = 0 mm. **(B)** OS in patients with CRM values between 0 and 30 mm. **(C)** OS in patients with CRM values larger than 30 mm.

We next assessed whether patients benefited less from chemotherapy as the CRM value increased, since higher CRM value meant a more thorough resection of the tumor. A Kaplan-Meier analysis was performed to investigate this assumption (Figure 3). Propensity score matching at 1:1 was performed prior to the Kaplan-Meier analysis, with stratification by age, AJCC stages, number of regional lymph nodes examined and positive regional lymph nodes, to minimize the influence of other independent prognostic factors. The benefit in the 3-year OS rate was 8.3, 10.1, and 9.2% for patients with a CRM value of 0 mm (40.5% for not receiving chemotherapy vs. 48.8% for receiving chemotherapy), between 0 and 30 mm (60.7 vs. 70.8%), and >30 mm (69.3 vs. 78.5%), respectively. Patients with a CRM value between 0 and 30 mm appeared to benefit most from chemotherapy compared with other groups.

### CRM Is Independent of Number of Regional Lymph Nodes Examined

So far, we have confirmed that patients with larger CRM values had better survival, and this can also be interpreted as patients who got more thorough resection of the tumor had better survival. Considering that number of regional lymph nodes examined is another indicator of how thorough the resection is, we wanted to go further into the relationship between the two risk factors. May it be possible that patients with larger CRM survive longer because they have more lymph nodes cut in the surgery? Previously we have confirmed that CRM positive patients tended to have fewer regional lymph nodes examined than CRM negative patients (Table 1), but after a propensity score match for factors including regional lymph nodes, CRM positivity is still negatively associated with OS (Figures 1A,B) and CSS Supplementary Figures 2A,B). Thus, in this part we paid more attention to the context of subgroups in CRM negative patients, that is, 0 < CRM ≤ 30 mm patients and CRM>30 mm patients. Supplementary Table 3 shows the baseline characteristics for 0 < CRM ≤ 30 mm and CRM>30 mm patients, from which we can see that patients with larger CRM values tended to have more regional lymph nodes examined and fewer positive regional lymph nodes. Again, a bipartite propensity score matching analysis was performed with stratification for age, AJCC stages, administration of chemotherapy, number of regional lymph nodes examined and positive regional lymph nodes, after which we can still find significantly better outcomes in both OS (Figures 1C,D) and CSS (Supplementary Figures 2C,D) for CRM>30 mm patients (*P* < 0.001). These results verify that although both being indicators of how thorough the resection is, CRM is independent of regional lymph nodes.

### Regional Lymph Nodes (≤ 13, >13) Can Be a Prognostic Indicator Instead of CRM for AJCC Stage I Patients

For AJCC stage I patients, X-tile program found no cutoffs with a statistically significant difference for CRM (Supplementary Figure 4A), but an optimal cutoff of 13 for regional lymph nodes (Supplementary Figure 5A). We then divided stage I patients into 2 cohorts of 0–13 lymph nodes and >13 lymph nodes, and do the X-tile analysis for CRM in each single cohort, but still no cutoffs with a statistically significant difference were defined (Supplementary Figures 5B,C). Thus, CRM is not suitable to be a prognostic predictor for stage I patients, but we can use number of regional lymph nodes examined instead.

## Discussion

The results of this study confirmed the significant unfavorable outcomes with CRM positivity, consistent with the findings by Amri et al. (13). Interestingly, the X-tile analysis revealed that aside from the 0 mm cutoff (diving the group into CRM-positive and CRM-negative), the remaining 30 mm cutoff value suggested that there was a survival difference among CRM-negative patients, and a CRM value larger than 30 mm indicated an enhanced survival. This cutoff value was tested for different AJCC stages separately. The survival of AJCC stage I patients seemed irrelevant with CRM value, while for stage II–IV patients, the best cutoff value was around 0 and 30 mm. These data are consistent with clinical experiences, since patients with early stage tumors tend to respond well to therapy and have a relatively good prognosis. Independent risk factors are shown in Supplementary Table 1, which includes chemotherapy but not radiation. A Kaplan-Meier analysis suggested that patients with a CRM value between 0 and 30 mm benefited most from chemotherapy. Possible explanations for this observation could be that patients with CRM values larger than 30 mm are less likely to have remnant tumor cells, and thus can benefit less from chemotherapy. In addition, the amount of circulating tumor cells in patients with CRM between 0 and 30 mm is much less than in CRM-positive patients. Consequently, chemotherapy could kill most circulating tumor cells in the 0–30 mm group, while only partially killing circulating tumor cells in the CRM positive group, leading to a relatively high response from chemotherapy in the 0–30 mm group. Besides, although both CRM and number of regional lymph nodes examined indicate how thorough the resection is, the effects of the two risk factors are independent. For stage I patients where CRM is not applicable, number of lymph nodes (0–13, >13) can be a good substitute as a prognostic predictor.

Currently, this is the largest retrospective study focusing on CRM in colon cancer, and is the first to investigate the survival differences among CRM-negative patients. We also discussed the possible deviations in optimal cutoff values from tumor progression, and whether CRM is a factor to consider when applying additional chemotherapy.

There are some limitations to the study. One of the limitations is within the SEER database itself. CRM is a factor associated with tumor recurrence, but the SEER database does not provide statistics for recurrence, so researchers are required to use OS and CSS instead. Secondly, as a large-scale retrospective study, the data is from different registries across the USA. Thus, the consistency in processing and interpretation of pathological specimens is not guaranteed, leading to bias caused by interrater variability. Thirdly, despite propensity score matching, significant differences still existed, such as in baseline characteristics of CRM-positive and CRM-negative groups. This might be attributed to the large sample size of the study cohort.

**Figure 4:**
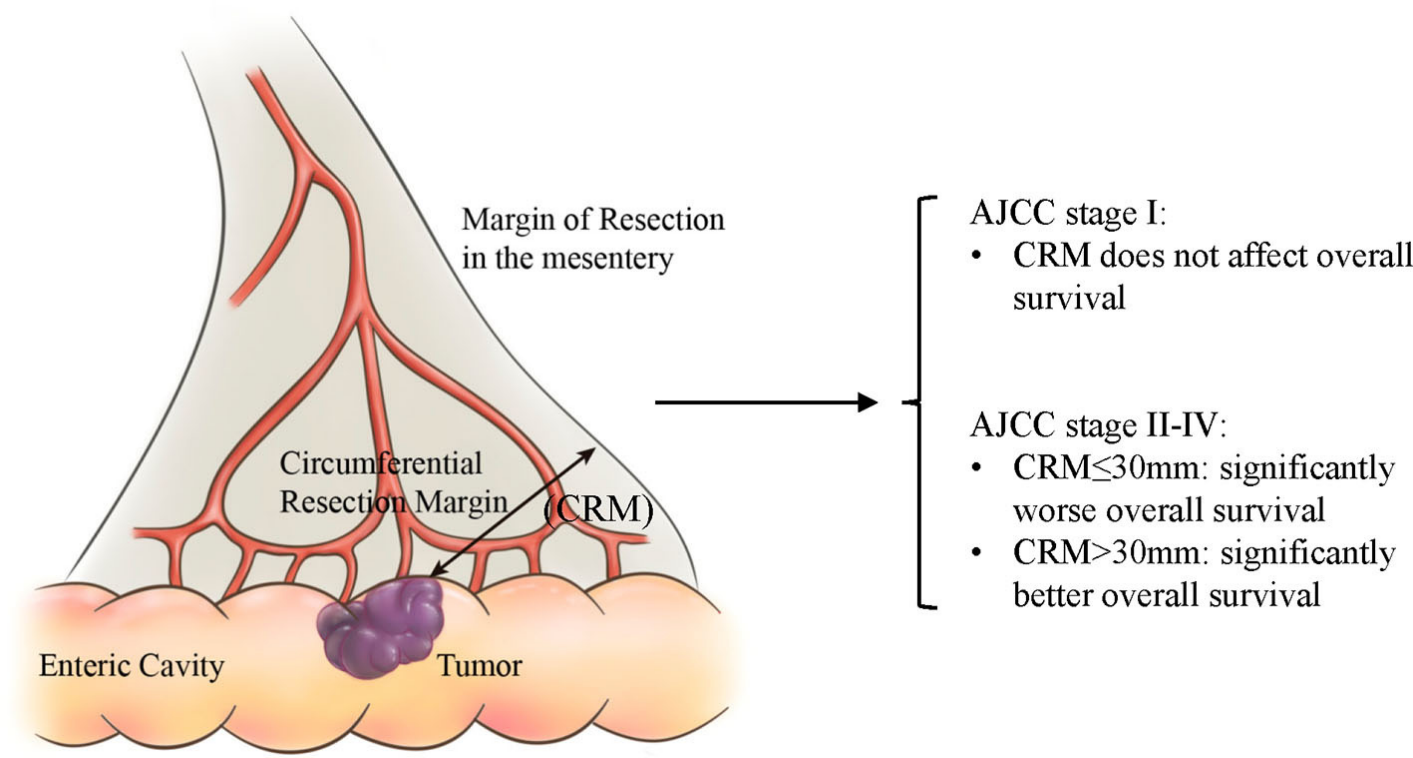
A schematic diagram of CRM in intraperitoneal colon cancer.

It is essential that, just like in rectal cancers, the CRM value of colon cancers be evaluated by pathologists in a standard format, which is advocated for by researchers like Efron (17). In ascending and descending colons, CRM refers to the distance to the margin of section in retroperitoneum, just like how CRM is measured in rectum cancer. In transverse colon and sigmoid colon, CRM refers to the distance to the margin of resection in mesentery, as is shown in Figure 4. Taylor et al. suggested that preoperative high-resolution magnetic resonance imaging (MRI) assessments of CRM status could estimate risk of local recurrence and survival better than AJCC-TNM based criteria (18). Also, by applying an MRI-based measurement, the frequency of neoadjuvant therapy and associated adverse side effects can be reduced without increasing the CRM positivity rate (19). Scott et al. demonstrated that poor differentiation, intravenous tumor invasion, peritoneal invasion, and lymph node metastasis are linked with a relatively higher rate of CRM positivity in colon cancer, and more aggressive surgery to obtain a clear margin benefited only a minority of patients (20). Nevertheless, we recommend appropriate expansion of the excision scope in order to obtain larger CRM values, which has been proven to be a favorable factor for survival.

## Conclusion

CRM positivity is a strong unfavorable indicator for the survival of colon cancer patients. Among CRM-negative patients, an improved outcome is expected if the CRM value is >30 mm. This cutoff value only applies to AJCC stage II–IV patients. For stage I patients, number of regional lymph nodes examined (0–13, >13) is a good substitute. Furthermore, patients with CRM values between 0 and 30 mm appear to benefit most from chemotherapy than the other two groups. We suggest appropriate expansion of the excision scope for advanced colon cancer patients to achieve larger CRM values.

## Author Contributions

X-YT, M-XH, S-QH, and YC were the major contributors in writing and revising the manuscript. X-MK, Y-YC, CL, Y-DH, Z-PL, and Y-TC performed the literature search. Y-TC, Z-JL, and X-YC participated in the design of the review and helped to finalize the manuscript. All authors read and approved the final manuscript.

## Conflict of Interest

The authors declare that the research was conducted in the absence of any commercial or financial relationships that could be construed as a potential conflict of interest.

## Supplementary Material

Supplementary Figure 1 Selection of eligible patients diagnosed with colon cancer between 2010 and 2015 with available Circumferential Resection Margin records from the Surveillance, Epidemiology, and End Results database. PSM, propensity score match.

Supplementary Figure 2 Kaplan-Meier curves for cancer-specific survival (CSS). **(A)** CSS in CRM-positive/negative patients. **(B)** CSS in CRM-positive/negative patients after 1:1 propensity score matching. **(C)** CSS in patients with CRM values of 0–30 and >30 mm. **(D)** CSS in patients with CRM values of 0–30 and >30 mm after 1:1 propensity score matching.

Supplementary Figure 3 Kaplan-Meier curves for cancer-specific survival (CSS) in patients with different AJCC stages stratified by CRM values. **(A)** CSS in stage I patients with CRM values 0/0–30/>30 mm. **(B)** CSS in stage II patients with CRM values 0/0–30 />30 mm. **(C)** CSS in stage III patients with CRM values 0/0–30/>30 mm. **(D)** CSS in stage IV patients with CRM values 0/0–30/>30 mm.

Supplementary Figure 4 X-tile analysis of survival data for optimal CRM cutoff values from records of patients stratified by AJCC stage. **(A)** Optimal cutoff values for stage I patients. **(B)** Optimal cutoff values for stage II patients. **(C)** Optimal cutoff values for stage III patients. **(D)** Optimal cutoff values for stage IV patients.

Supplementary Figure 5 X-tile analysis of survival data for optimal cutoffs for number of regional lymph nodes examined and CRM from records of stage I patients. **(A)** Optimal cutoff value for number of regional lymph nodes examined for stage I patients. **(B)** Optimal cutoff values for CRM for stage I patients who had 0–13 regional lymph nodes examined. **(C)** Optimal cutoff values for CRM for stage I patients who had >13 regional lymph nodes examined.

Supplementary Table 1 Univariable and multivariable Cox proportional hazard analysis of overall survival for all patients.

Supplementary Table 2 Univariate and multivariate Cox proportional hazard analysis of cancer-specific survival for all patients.

Supplementary Table 3 Baseline characteristics for 0 < CRM ≤ 30 mm and CRM>30 mm patients prior to and after propensity score matching.
