# Supplementary figures and images for: The Circumferential Resection Margin Is a Prognostic Predictor in Colon Cancer
Source: Front Oncol. 2020 Jun 26;10:927. doi: 10.3389/fonc.2020.00927 (PMC7332859; doi:10.3389/fonc.2020.00927)

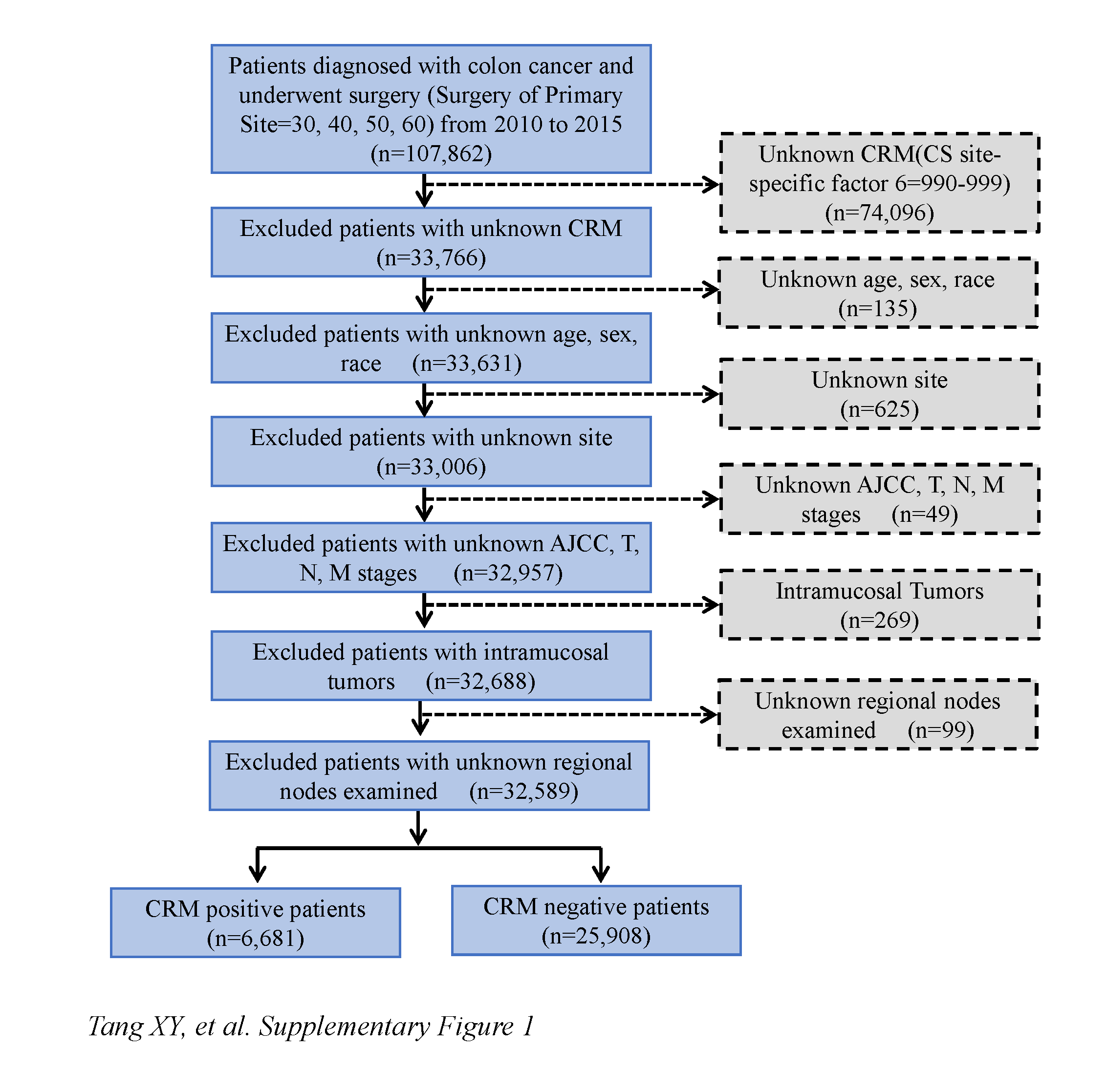

Supplement: Supplementary Figure 1 — Selection of eligible patients diagnosed with colon cancer between 2010 and 2015 with available Circumferential Resection Margin records from the Surveillance, Epidemiology, and End Results database. PSM, propensity score match. [file Image_1.TIFF]

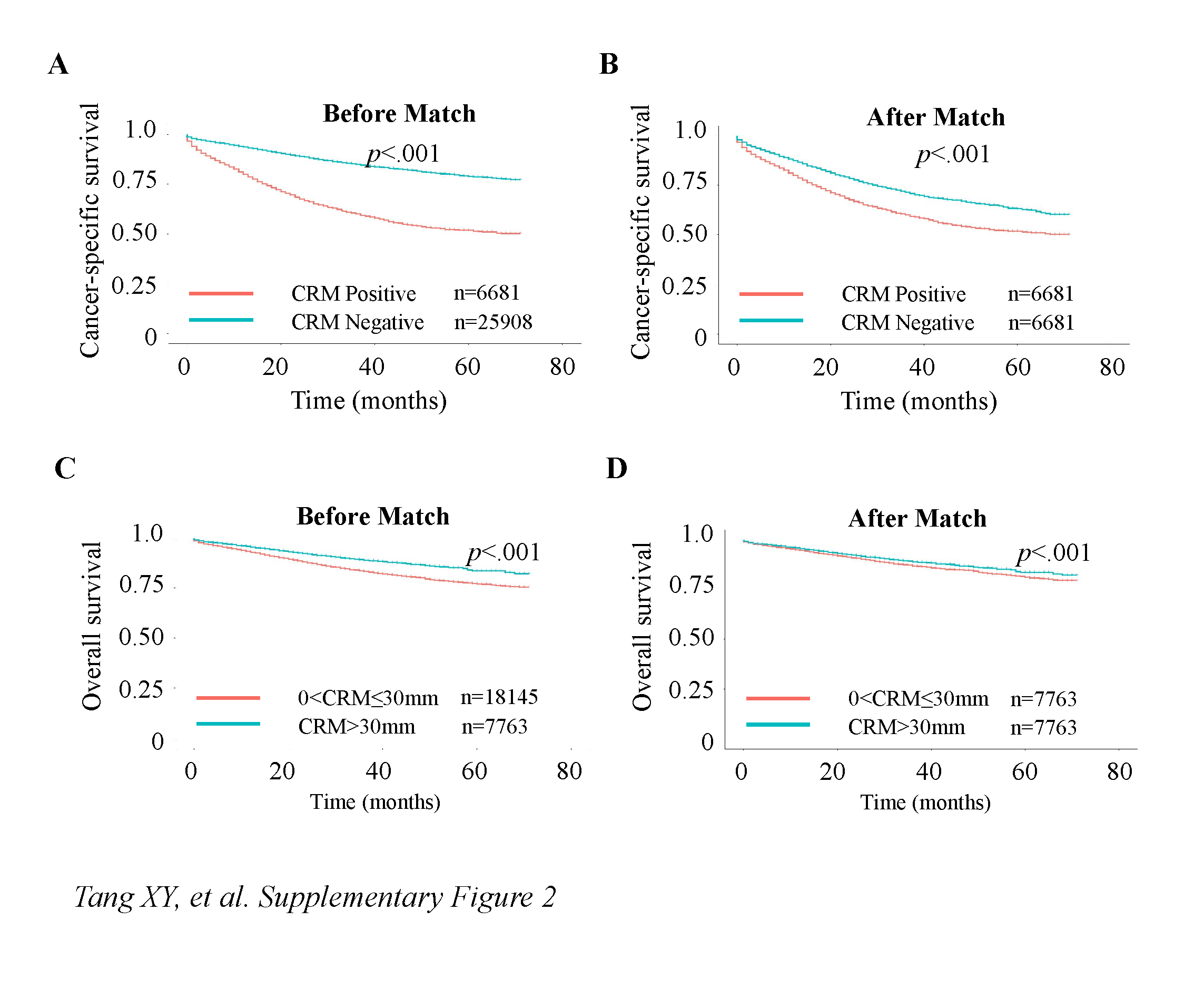

Supplement: Supplementary Figure 2 — Kaplan-Meier curves for cancer-specific survival (CSS). (A) CSS in CRM-positive/negative patients. (B) CSS in CRM-positive/negative patients after 1:1 propensity score matching. (C) CSS in patients with CRM values of 0–30 and >30 mm. (D) CSS in patients with CRM values of 0–30 and >30 mm after 1:1 propensity score matching. [file Image_2.TIFF]

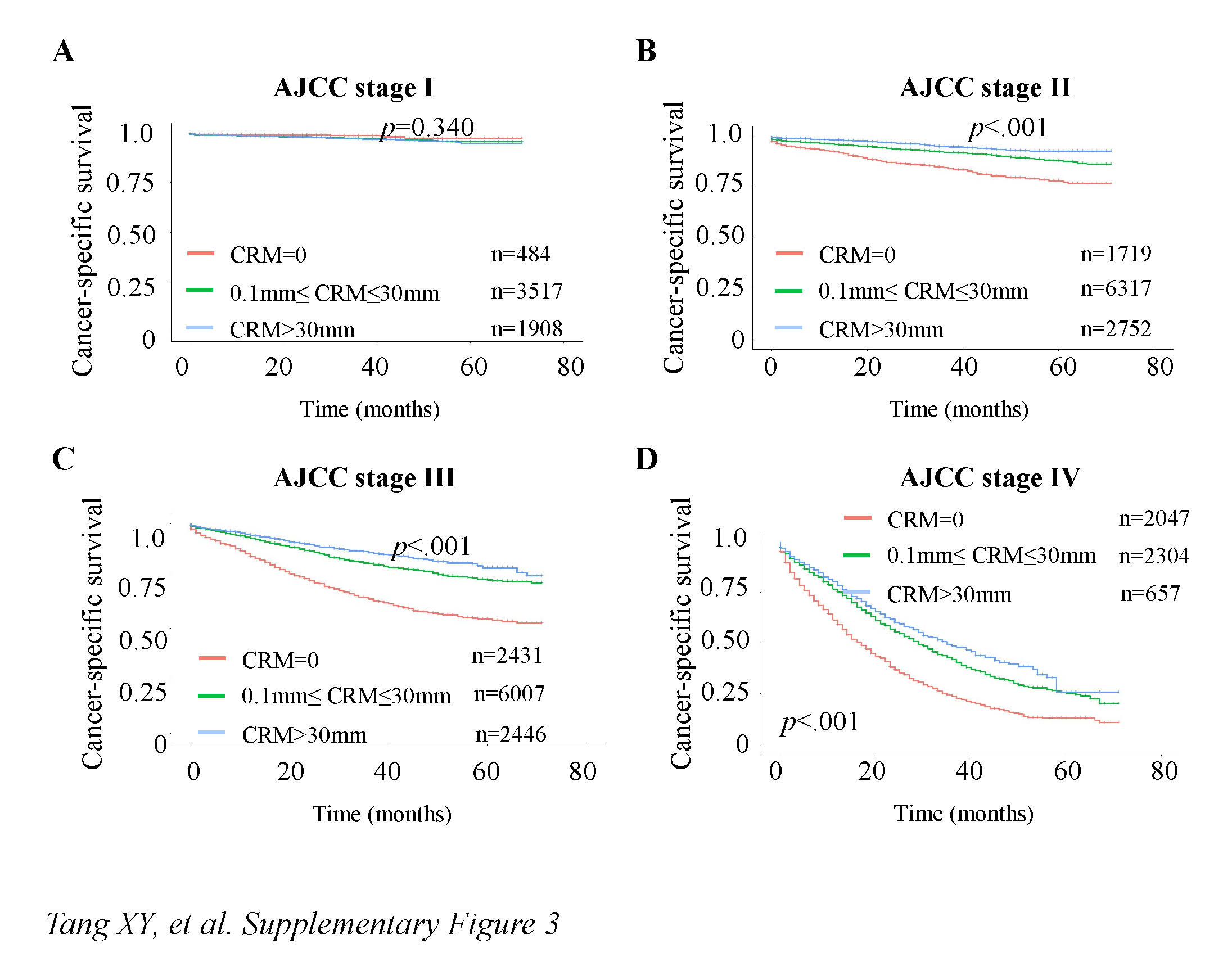

Supplement: Supplementary Figure 3 — Kaplan-Meier curves for cancer-specific survival (CSS) in patients with different AJCC stages stratified by CRM values. (A) CSS in stage I patients with CRM values 0/0–30/>30 mm. (B) CSS in stage II patients with CRM values 0/0–30 />30 mm. (C) CSS in stage III patients with CRM values 0/0–30/>30 mm. (D) CSS in stage IV patients with CRM values 0/0–30/>30 mm. [file Image_3.TIFF]

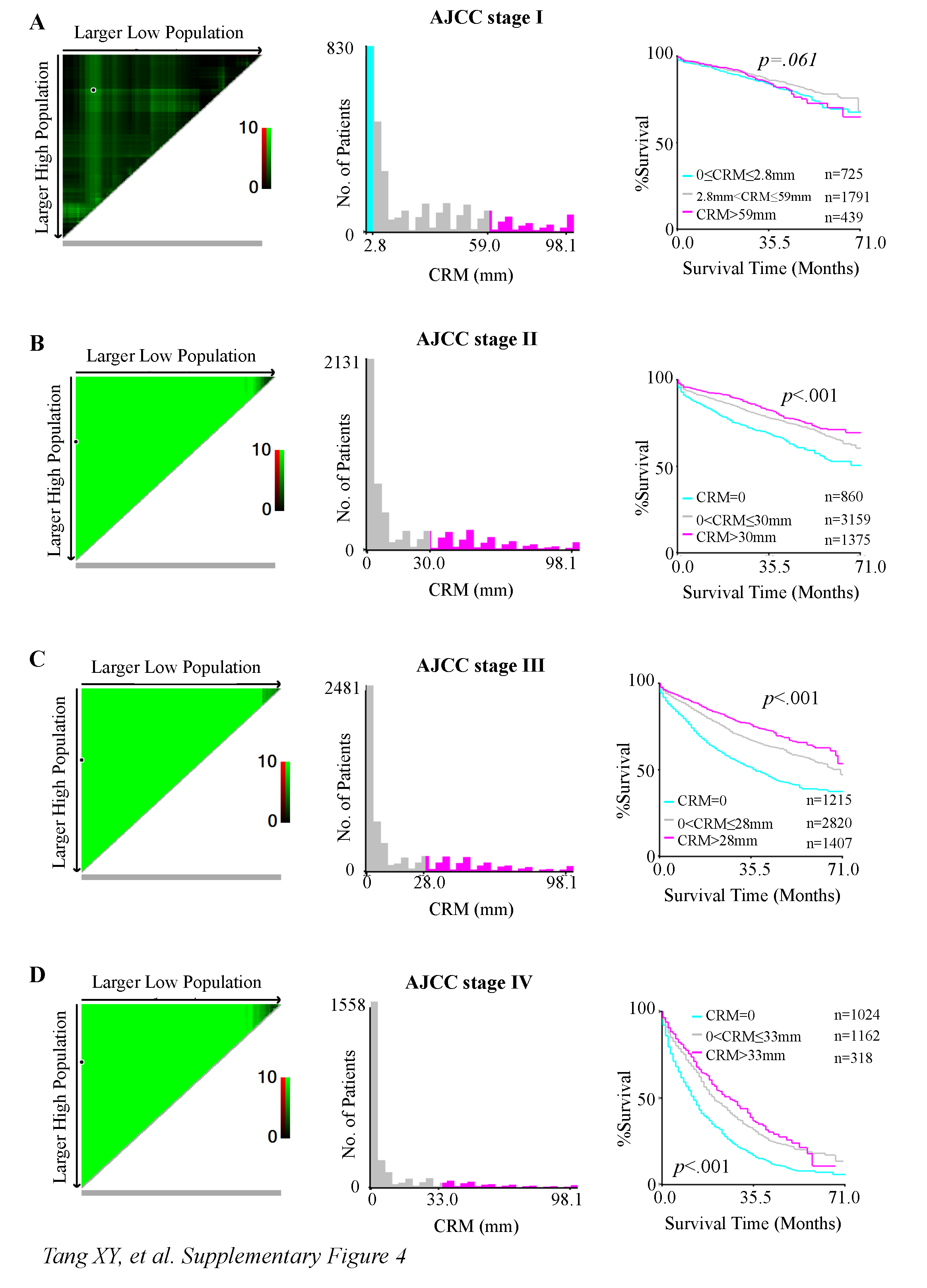

Supplement: Supplementary Figure 4 — X-tile analysis of survival data for optimal CRM cutoff values from records of patients stratified by AJCC stage. (A) Optimal cutoff values for stage I patients. (B) Optimal cutoff values for stage II patients. (C) Optimal cutoff values for stage III patients. (D) Optimal cutoff values for stage IV patients. [file Image_4.TIFF]

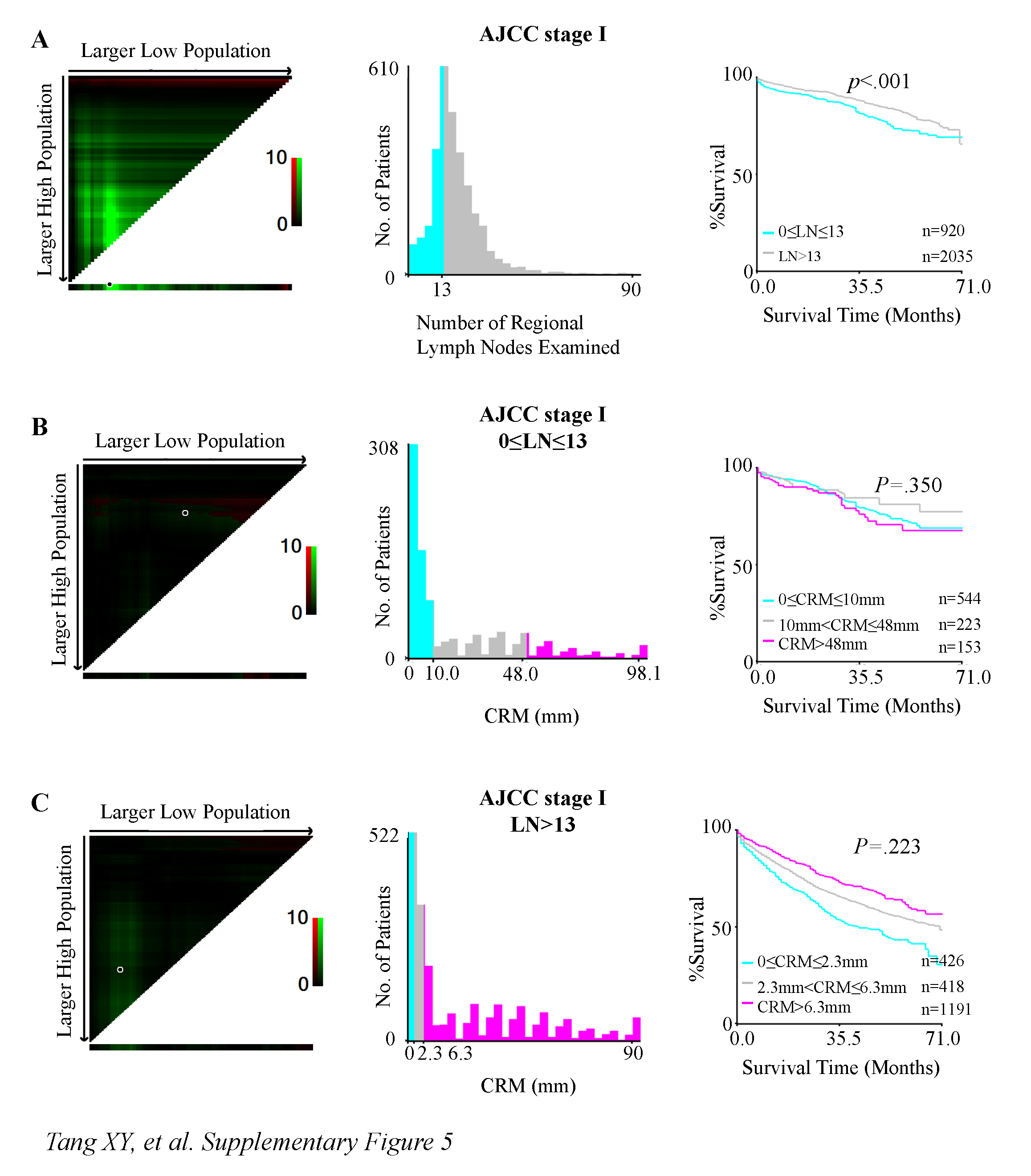

Supplement: Supplementary Figure 5 — X-tile analysis of survival data for optimal cutoffs for number of regional lymph nodes examined and CRM from records of stage I patients. (A) Optimal cutoff value for number of regional lymph nodes examined for stage I patients. (B) Optimal cutoff values for CRM for stage I patients who had 0–13 regional lymph nodes examined. (C) Optimal cutoff values for CRM for stage I patients who had >13 regional lymph nodes examined. [file Image_5.TIFF]
