# Supplementary material for: The Circumferential Resection Margin Is a Prognostic Predictor in Colon Cancer
Source: Front Oncol. 2020 Jun 26;10:927. doi: 10.3389/fonc.2020.00927 (PMC7332859; doi:10.3389/fonc.2020.00927)
Supplement: Supplementary Table 1 — Univariable and multivariable Cox proportional hazard analysis of overall survival for all patients. [file Table_1.docx]

**Supplementary Table 1**. Univariable and multivariable Cox proportional hazard analysis of overall survival for all patients.

|  | **Univariate analysis of OS** | | | **Multivariate analysis of OS** | | |
| --- | --- | --- | --- | --- | --- | --- |
| **Variables** | **Hazard Ratio** | **95%CI** | ***p*** | **Hazard Ratio** | **95%CI** | ***p*** |
| **Age** |  |  |  |  |  |  |
| <65 | 1 | - | - | 1 | - | - |
| ≥65 | 1.86 | 1.77-1.94 | <.001 | 1.88 | 1.79-1.98 | <.001 |
| **Sex** |  |  |  |  |  |  |
| Male | 1 | - | - | - | - | - |
| Female | 0.97 | 0.93-1.01 | .114 | - | - | - |
| **Race** |  |  |  |  |  |  |
| White | 1 | - | - | 1 | - | - |
| Black | 1.00 | 0.94-1.07 | .917 | 1.06 | 0.99-1.13 | .101 |
| Other (American Indian/AK Native, Asian/Pacific Islander) | 0.81 | 0.75-0.88 | <.001 | 0.86 | 0.79-0.93 | <.001 |
| **Year** |  |  |  |  |  |  |
| 2010 | 1 | - | - | 1 | - | - |
| 2011 | 0.94 | 0.88-1.00 | .046 | 1.02 | 0.96-1.09 | .485 |
| 2012 | 0.95 | 0.89-1.01 | .122 | 1.04 | 0.98-1.11 | .196 |
| 2013 | 0.96 | 0.90-1.03 | .237 | 1.09 | 1.02-1.17 | .013 |
| 2014 | 0.86 | 0.79-0.93 | <.001 | 1.01 | 0.93-1.09 | .895 |
| 2015 | 0.74 | 0.66-0.82 | <.001 | 0.90 | 0.81-1.00 | .048 |
| **AJCC** |  |  |  |  |  |  |
| I | 1 | - | - | 1 | - | - |
| II | 1.61 | 1.48-1.74 | <.001 | 0.94 | 0.80-1.10 | .418 |
| III | 2.52 | 2.34-2.73 | <.001 | 1.74 | 1.45-2.08 | <.001 |
| IV | 7.64 | 7.06-8.25 | <.001 | 4.92 | 4.11-5.88 | <.001 |
| **T** |  |  |  |  |  |  |
| T1 | 1 | - | - | 1 | - | - |
| T2 | 1.32 | 1.16-1.50 | <.001 | 1.31 | 1.15-1.50 | <.001 |
| T3 | 2.47 | 2.21-2.76 | <.001 | 2.16 | 1.82-2.57 | <.001 |
| T4 | 5.72 | 5.11-6.39 | <.001 | 3.35 | 2.81-3.98 | <.001 |
| **N** |  |  |  |  |  |  |
| N0 | 1 | - | - | 1 | - | - |
| N1 | 1.78 | 1.69-1.87 | <.001 | 1.04 | 0.88-1.24 | .626 |
| N2 | 3.49 | 3.33-3.67 | <.001 | 1.38 | 1.15-1.66 | .001 |
| **M** |  |  |  |  |  |  |
| M0 | 1 | - | - | 1 | - | - |
| M1 | 4.21 | 4.04-4.40 | <.001 | NA | NA-NA | NA |
| **Site** |  |  |  |  |  |  |
| Right Colon | 1 | - | - | 1 | - | - |
| Left Colon | 0.84 | 0.80-0.88 | <.001 | 0.89 | 0.85-0.94 | <.001 |
| **Histology** |  |  |  |  |  |  |
| Adenocarcinoma | 1 | - | - | 1 | - | - |
| Non-adenocarcinoma | 0.73 | 0.69-0.78 | <.001 | 1.01 | 0.95-1.07 | .708 |
| **Surgery** |  |  |  |  |  |  |
| Partial colectomy | 1 | - | - | 1 | - | - |
| Subtotal/Hemicolectomy | 1.14 | 1.09-1.19 | <.001 | 1.04 | 0.99-1.10 | .089 |
| Total colectomy | 1.51 | 1.31-1.72 | <.001 | 1.6 | 1.40-1.84 | <.001 |
| Total proctocolectomy | 0.95 | 0.64-1.42 | .818 | 1.42 | 0.96-2.10 | .083 |
| **Radiation** |  |  |  |  |  |  |
| No/Unknown | 1 | - | - | 1 | - | - |
| Yes | 1.18 | 1.02-1.35 | .022 | 1.13 | 0.98-1.30 | .087 |
| **Chemotherapy** |  |  |  |  |  |  |
| No/Unknown | 1 | - | - | 1 | - | - |
| Yes | 0.87 | 0.83-0.91 | <.001 | 0.36 | 0.34-0.38 | <.001 |
| **CRM** |  |  |  |  |  |  |
| 0 | 1 | - | - | 1 | - | - |
| 0<CRM≤30mm | 0.48 | 0.45-0.50 | <.001 | 0.80 | 0.76-0.84 | <.001 |
| CRM>30mm | 0.34 | 0.32-0.36 | <.001 | 0.67 | 0.62-0.71 | <.001 |
| **Regional LN Examined** |  |  |  |  |  |  |
| 0 | 1 | - | - | 1 | - | - |
| LN<12 | 1.07 | 0.83-1.39 | .603 | 0.78 | 0.60-1.01 | .059 |
| 12≤LN<24 | 0.71 | 0.55-0.92 | .009 | 0.54 | 0.41-0.70 | <.001 |
| LN≥24 | 0.54 | 0.42-0.70 | <.001 | 0.41 | 0.31-0.53 | <.001 |
| **Regional LN Positive** |  |  |  |  |  |  |
| No/Unknown | 1 | - | - | 1 | - | - |
| LN<6 | 1.87 | 1.79-1.96 | <.001 | 1.11 | 0.96-1.28 | .144 |
| 6≤LN<12 | 3.55 | 3.34-3.77 | <.001 | 1.36 | 1.16-1.61 | <.001 |
| LN≥12 | 5.53 | 5.13-5.97 | <.001 | 2.15 | 1.81-2.55 | <.001 |
| No LN Examined | 2.22 | 1.72-2.88 | <.001 | NA | NA-NA | NA |
