# Supplementary material for: The Circumferential Resection Margin Is a Prognostic Predictor in Colon Cancer
Source: Front Oncol. 2020 Jun 26;10:927. doi: 10.3389/fonc.2020.00927 (PMC7332859; doi:10.3389/fonc.2020.00927)
Supplement: Supplementary Table 2 — Univariate and multivariate Cox proportional hazard analysis of cancer-specific survival for all patients. [file Table_2.docx]

**Supplementary Table 2**. Univariate and multivariate Cox proportional hazard analysis of cancer-specific survival for all patients.

|  | **Univariate analysis of CSS** | | | | | | **Multivariate analysis of CSS** | | | | | |
| --- | --- | --- | --- | --- | --- | --- | --- | --- | --- | --- | --- | --- |
| **Variables** | **Hazard Ratio** | | **95%CI** | | ***p*** | | **Hazard Ratio** | | **95%CI** | | ***p*** | |
| **Age** |  | |  | |  | |  | |  | |  | |
| <65 | 1 | | - | | - | | 1 | | - | | - | |
| ≥65 | 1.14 | | 1.08-1.21 | | <.001 | | 1.34 | | 1.26-1.43 | | <.001 | |
| **Sex** |  | |  | |  | |  | |  | |  | |
| Male | 1 | | - | | - | | - | | - | | - | |
| Female | 1 | | 0.94-1.05 | | .915 | | - | | - | | - | |
| **Race** |  | |  | |  | |  | |  | |  | |
| White | 1 | | - | | - | | 1 | | - | | - | |
| Black | 1.15 | | 1.06-1.25 | | .001 | | 1.12 | | 1.03-1.22 | | .009 | |
| Other (American Indian/AK Native, Asian/Pacific Islander) | 0.96 | | 0.87-1.06 | | .384 | | 0.95 | | 0.86-1.05 | | .319 | |
| **Year** |  | |  | |  | |  | |  | |  | |
| 2010 | 1 | | - | | - | | 1 | | - | | - | |
| 2011 | 0.99 | | 0.92-1.08 | | .891 | | 1.09 | | 1.01-1.19 | | .038 | |
| 2012 | 0.96 | | 0.88-1.04 | | .332 | | 1.07 | | 0.98-1.17 | | .137 | |
| 2013 | 0.97 | | 0.88-1.06 | | .489 | | 1.14 | | 1.04-1.25 | | .006 | |
| 2014 | 0.90 | | 0.81-1.00 | | .044 | | 1.07 | | 0.96-1.18 | | .217 | |
| 2015 | 0.74 | | 0.64-0.85 | | <.001 | | 0.93 | | 0.80-1.07 | | .307 | |
| **AJCC** |  | |  | |  | |  | |  | |  | |
| I | 1 | | - | | - | | 1 | | - | | - | |
| II | 3.21 | | 2.68-3.84 | | <.001 | | 1.34 | | 1.02-1.76 | | .038 | |
| III | 8.12 | | 6.84-9.65 | | <.001 | | 3.63 | | 2.72-4.86 | | <.001 | |
| IV | 34.08 | | 28.73-40.42 | | <.001 | | 12.88 | | 9.65-17.18 | | <.001 | |
| **T** |  | |  | |  | |  | |  | |  | |
| T1 | 1 | | - | | - | | 1 | | - | | - | |
| T2 | 1.99 | | 1.48-2.67 | | <.001 | | 1.84 | | 1.37-2.49 | | <.001 | |
| T3 | 7.44 | | 5.73-9.65 | | <.001 | | 3.76 | | 2.72-5.19 | | <.001 | |
| T4 | 22.85 | | 17.61-29.65 | | <.001 | | 6.24 | | 4.51-8.65 | | <.001 | |
| **N** |  | |  | |  | |  | |  | |  | |
| N0 | 1 | | - | | - | | 1 | | - | | - | |
| N1 | 3.06 | | 2.84-3.30 | | <.001 | | 1.03 | | 0.83-1.28 | | .76 | |
| N2 | 7.18 | | 6.69-7.70 | | <.001 | | 1.43 | | 1.13-1.80 | | .003 | |
| **M** |  | |  | |  | |  | |  | |  | |
| M0 | 1 | | - | | - | | 1 | | - | | - | |
| M1 | 7.51 | | 7.1-7.94 | | <.001 | | NA | | NA-NA | | NA | |
| **Site** |  | |  | |  | |  | |  | |  | |
| Right Colon | 1 | | - | | - | | 1 | | - | | - | |
| Left Colon | 0.94 | | 0.89-1.00 | | .045 | | 0.91 | | 0.85-0.97 | | .006 | |
| **Histology** |  | |  | |  | |  | |  | |  | |
| Adenocarcinoma | 1 | | - | | - | | 1 | | - | | - | |
| Non-adenocarcinoma | 0.64 | | 0.59-0.69 | | <.001 | | 1.05 | | 0.96-1.14 | | .278 | |
| **Surgery** |  | |  | |  | |  | |  | |  | |
| Partial colectomy | 1 | | - | | - | | 1 | | - | | - | |
| Subtotal/Hemicolectomy | 1.10 | | 1.04-1.17 | | .001 | | 1.06 | | 0.99-1.13 | | .097 | |
| Total colectomy | 1.23 | | 1.01-1.50 | | .043 | | 1.35 | | 1.10-1.65 | | .004 | |
| Total proctocolectomy | 0.96 | | 0.57-1.62 | | .879 | | 1.43 | | 0.84-2.42 | | .187 | |
| **Radiation** |  | |  | |  | |  | |  | |  | |
| No/Unknown | 1 | | - | | - | | 1 | | - | | - | |
| Yes | 1.52 | | 1.28-1.79 | | <.001 | | 1.17 | | 0.99-1.39 | | .066 | |
| **Chemotherapy** |  | |  | |  | |  | |  | |  | |
| No/Unknown | 1 | | - | | - | | 1 | | - | | - | |
| Yes | 1.41 | | 1.33-1.48 | | <.001 | | 0.40 | | 0.37-0.42 | | <.001 | |
| **CRM** |  | |  | |  | |  | |  | |  | |
| 0 | 1 | | - | | - | | 1 | | - | | - | |
| 0<CRM≤30mm | 0.36 | | 0.34-0.38 | | <.001 | | 0.74 | | 0.70-0.79 | | <.001 | |
| CRM>30mm | 0.23 | | 0.21-0.25 | | <.001 | | 0.60 | | 0.55-0.66 | | <.001 | |
| **Regional LN Examined** |  | |  | |  | |  | |  | |  | |
| 0 | 1 | - | | - | | 1 | | - | | - | |  |
| LN<12 | 1.19 | 0.82-1.72 | | .361 | | 0.97 | | 0.67-1.41 | | .872 | |  |
| 12≤LN<24 | 0.79 | 0.55-1.14 | | .207 | | 0.68 | | 0.47-0.99 | | .042 | |  |
| LN≥24 | 0.61 | 0.42-0.88 | | .008 | | 0.50 | | 0.34-0.72 | | <.001 | |  |
| **Regional LN Positive** |  |  | |  | |  | |  | |  | |  |
| No/Unknown | 1 | - | | - | | 1 | | - | | - | |  |
| LN<6 | 3.21 | 2.99-3.44 | | <.001 | | 1.14 | | 0.94-1.37 | | .175 | |  |
| 6≤LN<12 | 7.01 | 6.46-7.61 | | <.001 | | 1.43 | | 1.16-1.76 | | .001 | |  |
| LN≥12 | 12.02 | 10.93-13.21 | | <.001 | | 2.36 | | 1.91-2.93 | | <.001 | |  |
| No LN Examined | 3.05 | 2.11-4.41 | | <.001 | | NA | | NA-NA | | NA | |  |
