# Supplementary material for: The Circumferential Resection Margin Is a Prognostic Predictor in Colon Cancer
Source: Front Oncol. 2020 Jun 26;10:927. doi: 10.3389/fonc.2020.00927 (PMC7332859; doi:10.3389/fonc.2020.00927)
Supplement: Supplementary Table 3 — Baseline characteristics for 0 < CRM ≤ 30 mm and CRM>30 mm patients prior to and after propensity score matching. [file Table_3.docx]

**Supplementary Table 3**. Baseline characteristics for 0<CRM≤30mm and CRM>30mm patients prior to and after propensity score matching.

|  | **Before matching** | | | **After matching** | | |
| --- | --- | --- | --- | --- | --- | --- |
| **Variables** | **0<CRM≤30**  **(n=18145)** | **CRM>30**  **(n=7763)** | ***p*** | **0<CRM≤30**  **(n=7763)** | **CRM>30**  **(n=7763)** | ***p*** |
|  | **n (%)** | **n (%)** |  | **n (%)** | **n (%)** |  |
| **Age** |  |  | .004 |  |  | 1 |
| <65 | 6659(36.7) | 2702(34.81) |  | 2702(34.81) | 2702(34.81) |  |
| ≥65 | 11486(63.3) | 5061(65.19) |  | 5061(65.19) | 5061(65.19) |  |
| **Sex** |  |  | .482 |  |  | .797 |
| Male | 9017(49.69) | 3820(49.21) |  | 3803(48.99) | 3820(49.21) |  |
| Female | 9128(50.31) | 3943(50.79) |  | 3960(51.01) | 3943(50.79) |  |
| **Race** |  |  | <.001 |  |  | .006 |
| White | 14449(79.63) | 6369(82.04) |  | 6214(80.05) | 6369(82.04) |  |
| Black | 2024(11.15) | 770(9.92) |  | 847(10.91) | 770(9.92) |  |
| Other (American Indian/AK Native, Asian/Pacific Islander) | 1672(9.21) | 624(8.04) |  | 702(9.04) | 624(8.04) |  |
| **Year of Diagnosis** |  |  | <.001 |  |  | <.001 |
| 2010 | 2639(14.54) | 649(8.36) |  | 1113(14.34) | 649(8.36) |  |
| 2011 | 2957(16.3) | 1071(13.8) |  | 1273(16.4) | 1071(13.8) |  |
| 2012 | 2966(16.35) | 1251(16.11) |  | 1208(15.56) | 1251(16.11) |  |
| 2013 | 2934(16.17) | 1449(18.67) |  | 1240(15.97) | 1449(18.67) |  |
| 2014 | 3290(18.13) | 1577(20.31) |  | 1439(18.54) | 1577(20.31) |  |
| 2015 | 3359(18.51) | 1766(22.75) |  | 1490(19.19) | 1766(22.75) |  |
| **AJCC^[[1]](#footnote-1)^** |  |  | <.001 |  |  | 1 |
| I | 3517(19.38) | 1908(24.58) |  | 1908(24.58) | 1908(24.58) |  |
| II | 6317(34.81) | 2752(35.45) |  | 2752(35.45) | 2752(35.45) |  |
| III | 6007(33.11) | 2446(31.51) |  | 2446(31.51) | 2446(31.51) |  |
| IV | 2304(12.7) | 657(8.46) |  | 657(8.46) | 657(8.46) |  |
| **T^a^** |  |  | <.001 |  |  | .001 |
| T1 | 1521(8.38) | 923(11.89) |  | 786(10.12) | 923(11.89) |  |
| T2 | 2779(15.32) | 1353(17.43) |  | 1434(18.47) | 1353(17.43) |  |
| T3 | 11156(61.48) | 4487(57.8) |  | 4602(59.28) | 4487(57.8) |  |
| T4 | 2689(14.82) | 1000(12.88) |  | 941(12.12) | 1000(12.88) |  |
| **N^a^** |  |  | <.001 |  |  | .680 |
| N0 | 10229(56.37) | 4771(61.46) |  | 7106(91.54) | 7106(91.54) |  |
| N1 | 4893(26.97) | 2006(25.84) |  | 657(8.46) | 657(8.46) |  |
| N2 | 3023(16.66) | 986(12.7) |  |  |  |  |
| **M^a^** |  |  | <.001 | 4774(61.5) | 4771(61.46) | 1 |
| M0 | 15841(87.3) | 7106(91.54) |  | 1972(25.4) | 2006(25.84) |  |
| M1 | 2304(12.7) | 657(8.46) |  | 1017(13.1) | 986(12.7) |  |
| **Site^[[2]](#footnote-2)^** |  |  | <.001 |  |  | <.001 |
| Right Colon | 11469(63.21) | 5264(67.81) |  | 5024(64.72) | 5264(67.81) |  |
| Left Colon | 6676(36.79) | 2499(32.19) |  | 2739(35.28) | 2499(32.19) |  |
| **Histology^[[3]](#footnote-3)^** |  |  | <.001 |  |  | <.001 |
| Adenocarcinoma | 14920(82.23) | 6078(78.29) |  | 6265(80.7) | 6078(78.29) |  |
| Non-adenocarcinoma | 3225(17.77) | 1685(21.71) |  | 1498(19.3) | 1685(21.71) |  |
| **Surgery** |  |  | .819 |  |  | .143 |
| Partial colectomy | 6859(37.8) | 2933(37.78) |  | 2850(36.71) | 2933(37.78) |  |
| Subtotal/Hemicolectomy | 10890(60.02) | 4661(60.04) |  | 4750(61.19) | 4661(60.04) |  |
| Total colectomy | 337(1.86) | 149(1.92) |  | 132(1.7) | 149(1.92) |  |
| Total proctocolectomy | 59(0.33) | 20(0.26) |  | 31(0.4) | 20(0.26) |  |
| **Radiation** |  |  | <.001 |  |  | .001 |
| No/Unknown | 17901(98.66) | 7705(99.25) |  | 7664(98.72) | 7705(99.25) |  |
| Yes | 244(1.34) | 58(0.75) |  | 99(1.28) | 58(0.75) |  |
| **Chemotherapy** |  |  | <.001 |  |  | 1 |
| No/Unknown | 11935(65.78) | 5326(68.61) |  | 5326(68.61) | 5326(68.61) |  |
| Yes | 6210(34.22) | 2437(31.39) |  | 2437(31.39) | 2437(31.39) |  |
| **Regional LN Examined** |  |  | <.001 |  |  | 1 |
| 0 | 81(0.45) | 11(0.14) |  | 11(0.14) | 11(0.14) |  |
| LN<12 | 1957(10.79) | 550(7.08) |  | 550(7.08) | 550(7.08) |  |
| 12≤LN<24 | 11309(62.33) | 4878(62.84) |  | 4878(62.84) | 4878(62.84) |  |
| LN≥24 | 4798(26.44) | 2324(29.94) |  | 2324(29.94) | 2324(29.94) |  |
| **Regional LN Positive** |  |  | <.001 |  |  | 1 |
| No/Unknown | 10525(58) | 4889(62.98) |  | 4889(62.98) | 4889(62.98) |  |
| LN<6 | 5664(31.22) | 2324(29.94) |  | 2324(29.94) | 2324(29.94) |  |
| 6≤LN<12 | 1376(7.58) | 423(5.45) |  | 423(5.45) | 423(5.45) |  |
| LN≥12 | 499(2.75) | 116(1.49) |  | 116(1.49) | 116(1.49) |  |
| No LN Examined | 81(0.45) | 11(0.14) |  | 11(0.14) | 11(0.14) |  |

1. [↑](#footnote-ref-1)
2. a American Joint Committee on Cancer. AJCC Cancer Staging Manual Seventh Edition. Springer. 2010[15].

   Right colon included cecum, ascending colon, hepatic flexure and transverse colon, while left colon included splenic flexure, descending colon and sigmoid colon. [↑](#footnote-ref-2)
3. Adenocarcinoma included International Classification of Diseases for Oncology = 8140, 8201, 8213, 8260, 8480, 8490, 8510. [↑](#footnote-ref-3)
